# Use of the α-mannosidase I inhibitor kifunensine allows the crystallization of apo CTLA-4 homodimer produced in long-term cultures of Chinese hamster ovary cells

**DOI:** 10.1107/S1744309111017672

**Published:** 2011-06-30

**Authors:** Chao Yu, Max Crispin, Andreas F.-P. Sonnen, David J. Harvey, Veronica T. Chang, Edward J. Evans, Christopher N. Scanlan, David I. Stuart, Robert J. C. Gilbert, Simon J. Davis

**Affiliations:** a Nuffield Department of Clinical Medicine, University of Oxford, John Radcliffe Hospital, Headington, Oxford OX3 9DS, England; b Oxford Glycobiology Institute, Department of Biochemistry, University of Oxford, Oxford OX1 1QU, England; c Division of Structural Biology, The Henry Wellcome Building for Genomic Medicine, University of Oxford, Roosevelt Drive, Oxford OX3 7BN, England

**Keywords:** kifunensine, glycoproteins, cytotoxic T-lymphocyte antigen 4

## Abstract

Glycoproteins present problems for structural analysis since they often have to be glycosylated in order to fold correctly and because their chemical and conformational heterogeneity generally inhibits crystallization. It is shown that the α-mannosidase I inhibitor kifunensine, which has previously been used for the purpose of glycoprotein crystallization in short-term (3–5 d) cultures, is apparently stable enough to be used to produce highly endoglycosidase H-sensitive glycoprotein in long-term (3–4 week) cultures of stably transfected Chinese hamster ovary (CHO) cells. Matrix-assisted laser desorption/ionization time-of-flight mass spectrometry-based analysis of the extracellular region of the cytotoxic T-lymphocyte antigen 4 (CTLA-4; CD152) homodimer expressed in long-term CHO cell cultures in the presence of kifunensine revealed that the inhibitor restricted CTLA-4 glycan processing to Man_9_GlcNAc_2_ and Man_5_GlcNAc_2_ structures. Complex-type glycans were undetectable, suggesting that the inhibitor was active for the entire duration of the cultures. Endoglycosidase treatment of the homodimer yielded protein that readily formed orthorhombic crystals with unit-cell parameters *a* = 43.9, *b* = 51.5, *c* = 102.9 Å and space group *P*2_1_2_1_2_1_ that diffracted to Bragg spacings of 1.8 Å. The results indicate that kifunensine will be effective in most, if not all, transient and long-term mammalian cell-based expression systems.

## Abstract 2

The α-mannosidase I inhibitor kifunensine inhibited N-glycan processing in long-term cultures of Chinese hamster ovary cells, allowing deglycosylation and crystallization of the homodimeric extracellular region of the inhibitory glycoprotein receptor CTLA-4 (CD152).

## 1. Introduction

Protein glycosylation generally inhibits the crystallization of glycoproteins, but is often required for their correct folding (reviewed by Davis & Crispin, 2011 ▶). Our initial solutions to the glycosylation problem involved the use of long-term Chinese hamster ovary (CHO) cell-based expression systems in which folding and initial glycosyl­ation of the glycoprotein were allowed to proceed normally but the subsequent processing of the N-linked glycans was restricted in a way that allowed their enzymatic removal from the purified glycoprotein with endoglycosidase H (Endo H; Davis *et al.*, 1993 ▶, 1995 ▶; Butters *et al.*, 1999 ▶). Endo H cleaves between the GlcNAc residues in the di-*N*-­acetylchitobiose core of oligomannose-type and hybrid-type N-­glycans, leaving single β-GlcNAc residues at each glycosylation sequon. To implement these methods in a high-throughput (*i.e.* structural-genomics-based) setting, we explored the use of glycosyl­ation processing inhibitors in cells that can be transiently transfected, such as human embryonic kidney (HEK) 293T cells (Chang *et al.*, 2007 ▶). This required the use of inhibitors that are likely to circumvent the endomannosidase-based shunt pathway present in HEK 293T cells, which currently provide the benchmark for high-level transient mammalian protein expression (Chang *et al.*, 2007 ▶). We found that glycoproteins expressed in 3–5 d cultures of HEK 293T cells in the presence of relatively low levels of the α-mannosidase I inhibitor kifunensine exhibited high levels of sensitivity to Endo H without compromising overall expression yields. Although the general effects of kifunensine and other alkaloid-like processing inhibitors have been very well established (Elbein, 1991 ▶), for the most part this information has come from biochemical analyses of relatively short-term cultures. Following culture for 12 d in the presence of kifunensine, recombinant human immunoglobulin expressed in CHO cells was shown to contain ‘mainly’ oligomannose-type N-glycans (van Berkel *et al.*, 2010 ▶), but the ability of kifunensine to efficiently inhibit glycan processing for periods beyond this was unknown.

Not all proteins of structural interest are amenable to high-throughput expression in transient cultures and our previous work with long-term stable expression systems involved the use of difficult-to-transfect cells or hard-to-obtain glycosylation inhibitors (Davis & Crispin, 2011 ▶). The apo form of the homodimeric extracellular region of human cytotoxic T-lymphocyte antigen 4 (CTLA-4; CD152), the product of the *CTLA4* gene and a critical modulator of human immune responses (Fife & Bluestone, 2008 ▶), refolds incorrectly from bacterial inclusion bodies (Sonnen *et al.*, 2010 ▶) and is usually expressed as an immunoglobulin Fc fusion protein to effect faithful dimerization, which compromises yields. We show that kifunensine is an effective inhibitor of N-glycan processing in long-term (3–4 week) cultures of CHO cells and use this method to obtain high-quality crystals of the apo CTLA-4 homodimer. Our results suggest that kifunensine will be effective in most, if not all, short-term and long-term mammalian cell expression systems. Analysis of the crystals of the CTLA-4 homodimer has provided key insights into the structural basis of triggering of this important receptor (Yu *et al.*, 2010 ▶).

## 2. Methods

### 2.1. Protein expression

Chimeric cDNA encoding, in the following order, residues 1–161 of the extracellular (ex) region of human CTLA-4, including the signal peptide sequence, a thrombin cleavage site, the heavy-chain constant domains 2 and 3 of murine IgG1 (Fc) and a C-terminal Lys-His_6_ tag was cloned into the pEE14 expression vector (Bebbington & Hentschell, 1987 ▶; Davis *et al.*, 1990 ▶). The expression vector encoding the chimeric CTLA-4-Fc-Lys-His_6_ construct (abbreviated to CTLA-4exFc) was then transfected into CHO-K1 cells followed by clone selection with 25 µ*M* methionine sulfoximine. Among the surviving clones, one expressed CTLA-4exFc at a level of approximately 15 mg l^−1^ and this was used for protein production in large-scale cultures (Cell Factories; Nunc, Roskilde, Denmark) in the presence of 10 µ*M* kifunensine (Toronto Research Chemicals, North York, Ontario, Canada) for up to three weeks following the addition of 2 m*M* sodium butyrate to boost expression.

### 2.2. Protein purification and crystallization

The CTLA-4exFc was harvested after 3–4 weeks of culture and the protein was extracted by metal-chelate chromatography using Ni–NTA agarose (Qiagen, West Sussex, England). The CTLA-4exFc was eluted from the Ni–NTA agarose with 250 m*M* imidazole in 20 m*M* Tris–HCl, 0.5 *M* NaCl pH 8.0 and further purified by size-exclusion chromatography (Superdex 200 HR 10/30 column; GE Healthcare, Amersham, England). Removal of the Fc from CTLA-4exFc, yielding residues 1–126 of the mature CTLA-4 polypeptide followed by the Leu-Val-Pro-Arg sequence from the cleaved thrombin site, was achieved by treating the protein with thrombin in 10 m*M* HEPES, 150 m*M* NaCl pH 7.4 at room temperature for 16 h. Non-Ni–NTA-agarose-bound (*i.e.* cleaved) CTLA-4ex homodimer was buffer-exchanged to 10 m*M* HEPES, 150 m*M* NaCl pH 7.4 and deglycosyl­ated with Endo H_f_ (New England Biolabs, Hitchin, England) at room temperature for 3 h. The deglycosylated Endo H_f_-treated CTLA-4ex homodimer was purified by lectin-affinity chromatography and gel filtration as previously described (Davis *et al.*, 1995 ▶).

Initial conditions for CTLA-4ex homodimer crystallization were screened using a sparse-matrix crystallization screening kit at 295 K (Hampton Research, Laguna Niguel, California, USA). Droplets (100 nl) of protein at 14.5 mg ml^−1^ in 10 m*M* HEPES, 150 m*M* NaCl pH 7.4 were mixed with 100 nl reservoir solution and set up in 96-well plates as described previously (Walter *et al.*, 2005 ▶). Following freezing of the crystals in glycerol/precipitant, diffraction data were collected on beamline I04 of the Diamond Light Source, England. Data were processed and scaled with the *HKL* suite (Otwinowski & Minor, 1997 ▶).

### 2.3. Glycan analysis and structural assignment

Glycans were released using protein *N*-glycanase (PNGase) F (Küster *et al.*, 1997 ▶) for analysis by positive-ion matrix-assisted laser desorption/ionization (MALDI) time-of-flight (TOF) mass spectrometry (MS) and negative-ion electrospray ionization (ESI) MS. Bands from Coomassie Blue-stained SDS–PAGE gels containing approximately 10 µg target glycoprotein were excised and eluted with alternating water/acetonitrile washes, dried and rehydrated with 30 µl 30 m*M* NaHCO_3_ pH 7.0 containing 100 units ml^−1^ of PNGase F (Prozyme, San Leandro, California, USA). The released N-linked glycans were eluted and cleaned with a Nafion 117 membrane (Börnsen *et al.*, 1995 ▶) prior to mass spectrometry. Positive-ion MALDI–TOF mass spectra were recorded with a Shimazu AXIMA TOF^2^ MALDI TOF/TOF mass spectrometer (Shimadzu-Kratos, Manchester, England) fitted with delayed extraction and a nitrogen laser (337 nm). Samples were prepared by adding 0.5 µl of an aqueous solution of the glycans to the matrix solution [0.3 µl of a saturated solution of recrystallized 2,5-dihydroxybenzoic acid in 1:1(*v*:*v*) acetonitrile:water containing a trace of sodium chloride] on the stainless-steel target plate and allowing it to dry at room temperature, followed by recrystallization from ethanol.

MALDI mass spectra of the glycans from the CTLA-4 glycoforms revealed compositional information by the identification of (*M* + Na)^+^ ions, whilst the structural assignment of the ions was achieved by negative-ion ESI MS using a Waters quadrupole time-of-flight (Q-­Tof) Ultima Global instrument (Waters MS Technologies, Manchester, England). For ESI MS, samples in 1:1(*v*:*v*) methanol:water were infused through Proxeon nanospray capillaries (Proxeon Biosystems, Odense, Denmark). The ion-source conditions were as follows: temperature, 393 K; nitrogen flow, 50 l h^−1^; infusion needle potential, 1.1 kV; cone voltage, 100 V; RF-1 voltage, 180 V. For MS/MS data acquisition, the parent ion was selected at low resolution to allow transmission of isotope peaks and was fragmented with argon at a pressure of 100 Pa. Instrument control, data acquisition and processing were performed with *MassLynx* software v.4.1 (Waters). Fragmentation spectra were analyzed as described previously (Harvey, 2005*a* ▶,*b* ▶,*c* ▶,*d* ▶; Harvey *et al.*, 2008 ▶). The nomenclature used follows that of Domon & Costello (1988 ▶) and is distinct from the established labels of the branches of the oligomannose-type glycans D1–D3.

## 3. Results

### 3.1. Expression and crystallization of CTLA-4ex from CHO-K1 cells cultured with kifunensine

**Figure 1:**
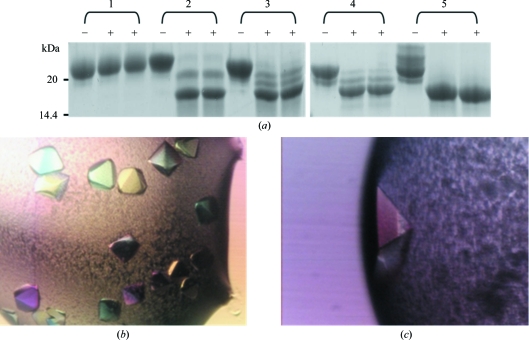
Deglycosylation and crystallization of the apo CTLA-4 homodimer. (*a*) Coomassie-stained SDS–polyacrylamide gel run under reducing conditions showing undigested (−) and Endo H-digested (+) CTLA-4ex expressed in wild-type and mutant CHO cells with and without glycan-processing inhibitors. Samples in the left and right lanes marked with (+) were Endo H-digested for 1 and 3 h, respectively, in order to confirm that after 1 h the reaction had proceeded to completion. Sample 1, CHO-K1 cells only; sample 2, CHO-K1 cells with 1.5 m*M* NB-DNJ; sample 3, CHO-K1 cells with 10 µ*M* kifunensine; sample 4, CHO Lec3.2.8.1 cells only; sample 5, CHO Lec3.2.8.1 cells with 0.5 m*M* NB-DNJ. In (*b*) crystals were grown in 25%(*w*/*v*) polyethylene glycol 1500 in 0.1 *M* sodium propionate/sodium cacodylate/Bis-Tris propane buffer pH 6.0 (Molecular Dimensions). These crystals were ∼100 × 100 × 100 µm in size. The crystal shown in (*c*) was grown in 0.2 *M* ammonium acetate, 25%(*w*/*v*) polyethylene glycol 1500, 0.1 *M* Bis-Tris pH 5.5 (Hampton Research). This crystal was ∼100 × 200 × 100 µm in size.

CTLA-4ex homodimer was prepared from the supernatants of long-term (3–4 week) cultures of CHO-K1 cells grown in the presence of kifunensine. Preliminary optimization utilizing a chimeric Fc-fusion protein containing the extracellular region of the programmed death 1 (PD-1) protein indicated that the lowest concentration of kifunensine ensuring largely complete Endo H-sensitivity following expression in CHO-K1 cells was 10 µ*M* (data not shown). To compare the sensitivity of CTLA-4ex generated in the presence of kifunensine *versus* other approaches, we also prepared CTLA-4ex from untreated CHO-K1 cells, from CHO-K1 cells treated with the α-glucosidase I inhibitor *N*-butyldeoxynojirimycin (NB-DNJ; Davis *et al.*, 1995 ▶), from mutant CHO-derived Lec3.2.8.1 cells whose ability to process glycans beyond the Man_5_GlcNAc_2_ stage is severely limited (Davis *et al.*, 1993 ▶) and from Lec3.2.8.1 cells treated with NB-DNJ (Butters *et al.*, 1999 ▶). Whereas the CHO-K1 cell-derived CTLA-4ex was completely resistant to Endo H, all other forms exhibited varying degrees of sensitivity, with protein expressed in Lec3.2.8.1 cells and in CHO-K1 cells, both with NB-DNJ, exhibiting the greatest and least sensitivity, respectively (Fig. 1 ▶ *a*). CTLA-4ex expressed in the presence of kifunensine, and in Lec3.2.8.1 cells only, exhibited similar intermediate levels of sensitivity. The sensitivity of CTLA-4ex expressed in CHO-K1 cells in the presence of kifunensine during 3–4 week cultures was comparable to that observed for proteins expressed in short-term (*i.e.* 2–3 d) cultures of HEK 293T cells in the presence of the inhibitor (Chang *et al.*, 2007 ▶).

Crystals of deglycosylated lectin-purified CTLA-4ex grew in a variety of conditions: (i) 25%(*w*/*v*) polyethylene glycol 1500 in 0.1 *M* sodium propionate/sodium cacodylate/Bis-Tris propane buffer pH 6.0; (ii) 0.2 *M* sodium dihydrogen phosphate, 25%(*w*/*v*) polyethylene glycol 3350; (iii) 30%(*w*/*v*) polyethylene glycol 6000, 0.1 *M* citrate pH 5.0 and (iv) 0.2 *M* ammonium acetate, 25%(*w*/*v*) polyethylene glycol 1500, 0.1 *M* Bis-Tris pH 5.5. Examples of crystals are shown in Figs. 1 ▶(*b*) and 1 ▶(*c*). The best diffraction observed was to 1.8 Å resolution, but the diffraction had a high background and contained a good number of split spots with high mosaicity. However, diffraction was improved by scaling up the drop volume from 200 nl to 2 µl and by using the traditional sitting-drop vapour-diffusion method (Harlos *et al.*, 1992 ▶). The self-rotation function and a Harker section of the native Patterson function clearly established that the space group is *P*2_1_2_1_2_1_ (data not shown). Data-collection and processing statistics are given in Table 1 ▶.

### 3.2. Effects of kifunensine on glycan processing in CHO-K1 cells

**Figure 2:**
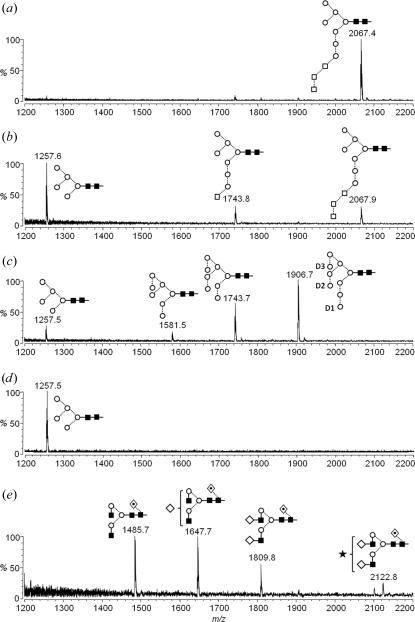
Positive-ion MALDI–TOF mass spectra of N-linked glycans from glycoforms of CTLA-4ex expressed in CHO-K1 cells with 1.5 m*M* NB-DNJ (*a*), CHO Lec3.2.8.1 cells with 0.5 m*M* NB-DNJ (*b*), CHO-K1 cells with 10 µ*M* kifunensine (*c*), CHO Lec3.2.8.1 cells (*d*) and CHO-K1 cells (*e*). A trace amount of putative Man_3_GlcNAc_2_ and Man_4_GlcNAc_2_ glycans was detected in the spectra of CHO Lec3.2.8.1 but was obscured by contaminating peaks (not shown). Symbols used for the structural formulae: diamonds, Gal; filled diamonds, GalNAc; filled squares, GlcNAc; circles, Man; stars, sialic acid; diamonds with dots in, Fuc. The linkage position is shown by the angle of the lines linking the sugar residues (vertical line, 2-link; forward slash, 3-link; horizontal line, 4-link; back slash, 6-link), whilst the anomericity is indicated by full lines for α-bonds and broken lines for β-bonds (Harvey *et al.*, 2009 ▶).

The glycans released from CTLA-4ex homodimers expressed under the five sets of conditions were analyzed by MALDI–TOF and ESI mass spectrometry. The MALDI mass spectrum for N-linked glycans from CTLA-4ex from CHO-K1 cells cultured with 1.5 m*M* NB-DNJ revealed a dominant peak at *m*/*z* 2067 corresponding to Hex_10_HexNAc_2_ (Fig. 2 ▶ *a*). This composition can be assigned to the specific isomer Glc_3_Man_7_GlcNAc_2_ by the collision-induced decomposition (CID) spectrum of the corresponding (*M* + Cl)^−^ ion, which revealed diagnostic ions for the trimannosyl 6-antennae (C_3α_ and C_3β_ at *m*/*z* 503.1 and the D and D − 18 ions at 647.2 and 629.2, respectively) and the 3-antennae (^1,3^A_5_ and ^2,4^A_5_ ions at *m*/*z* 707.2 and ^1,3^A_6_ and ^2,4^A_6_ at *m*/*z* 869.2) (Supplementary Fig. S1*a* 1). This structure is consistent with the almost complete inhibition of processing by α-­glucosidase I achieved at 1.5 m*M* and matches that previously reported for glycoproteins expressed in CHO-K1 cells with high levels of NB-DNJ (Butters *et al.*, 1999 ▶; Crispin *et al.*, 2006 ▶). The ^2,4^A_8_, B_8_ and ^2,4^A_8_ ions and corresponding ions in the other spectra shown in Supplementary Fig. S1(*a*)^1^ are diagnostic for the mannosyl-chito­biose trisaccharide core and show no substitutions in this region of the molecule.

For glycans derived from CHO Lec3.2.8.1 cells in the presence of 0.5 m*M* NB-DNJ (Fig. 2 ▶ *b*) the dominant ion at *m*/*z* 2067.9 gave a CID spectrum indicating Glc_3_Man_7_GlcNAc_2_ as above. The corresponding spectrum of the ion at *m*/*z* 1257.6 was identical to that from a reference sample of Man_5_GlcNAc_2_. The spectrum of the ion at *m*/*z* 1743.8 corresponding to Hex_8_HexNAc_2_ is shown in Supplementary Fig. S1(*b*)^1^. B_3β_, C_3β_, ^0,3^A_7_, D − 18 and D ions at *m*/*z* 485, 503, 575, 629 and 647, respectively, showed the presence of the Man_3_-containing 6-­antenna. The other four hexose residues must therefore comprise the 3-antenna and consist of Glc_1_Man_3_. This conclusion was supported by the ions at *m*/*z* 545, 383 and 323, which were specific to the 3-antenna and 2 × 162 mass units lower than their counterparts in the spectrum of Glc_3_Man_7_GlcNAc_2_.

In the MALDI–TOF spectrum for glycans from CHO-K1 cells in the presence of 10 µ*M* kifunensine (Fig. 2 ▶ *c*), compounds producing the ions at *m*/*z* 1905.7 and 1257.5 gave CID spectra showing the normal Man_9_GlcNAc_2_ and Man_5_GlcNAc_2_ structures. The spectrum of Man_9_GlcNAc_2_ is shown in Supplementary Fig. S1(*c*)^1^. B_3β_, C_3β_, ^0,3^A_7_, D − 18 and D ions at *m*/*z* 809, 827, 899, 953 and 971, respectively, confirmed the Man_5_ structure of the 6-antenna. The corresponding CID spectrum of the compound with the composition Hex_8_HexNAc_2_ (*m*/*z* 1743.7 in the MALDI–TOF spectrum) is shown in Supplementary Fig. S1(*d*)^1^. Its CID spectrum contained B<_3β_, C_3β_, ^0,3^A_7_, D − 18 and D ions at the same masses as those in the spectrum of Man_9_GlcNAc_2_, indicating a Man_5_-containing 6-antenna and showing that a mannose was missing from the 3-antenna. The isomeric *D*1,*D*3 Man_8_GlcNAc_2_ that is the normal product of the first exomannosidase cleavage gave the spectrum shown in Supplementary Fig. S1(*e*)^1^. The presence of one fewer mannose residue in the 6-antenna is reflected by the masses of the B_3β_, C_3β_, ^0,3^A_7_, D − 18 and D ions, which are 162 units lower than in the spectrum of the isomer shown in Supplementary Fig. S1(*d*)^1^. Complex-type structures were undetectable in the spectrum from kifunensine-treated cells. The MALDI–TOF spectrum for glycans released from CTLA-4ex expressed in CHO Lec3.2.8.1 cells (Fig. 2 ▶ *d*) revealed the production of only Man_5_GlcNAc_2_, whereas the spectrum for protein from untreated CHO-K1 cells (Fig. 2 ▶ *e*) showed complex-type glycans, explaining its unreactivity towards Endo H.

## 4. Discussion

Since kifunensine targets highly conserved class I mannosidases downstream of the endomannosidase-dependent shunt pathway, our expectation was that it might be effective in a number of mammalian-cell-based expression systems (Chang *et al.*, 2007 ▶; Nettleship *et al.*, 2009 ▶). It was not obvious, however, that kifunensine was sufficiently stable in very long-term (*e.g.* 3–4 week) stable cultures *versus* short-term (2–­3 d) transient cultures to affect the glycan processing of all the glycoprotein produced during extended culture. However, we succeeded in producing glycoprotein that could be very efficiently deglycosylated with Endo H even from cultures kept for as long as four weeks. The glycan analysis was notable for the complete absence of detectable complex-type glycans in the sample prepared in the presence of kifunensine, suggesting either that the inhibitor is stable for the entire period of the culture or that the cells only express protein for a brief period during which the inhibitor is active. Analysis of the kinetics of expression of other proteins, in which the cells secrete the heterologous gene product for the lifetime of the culture (S. J. Davis *et al.*, unpublished work), supports the former possibility. Although we did not directly examine the effect of kifunensine on the levels of expression of CTLA-4ex, we previously showed that transiently transfected HEK 293T cells treated with the inhibitor produced 30% more protein than untreated cells, presumably owing to effects on ER-associated degradation (Chang *et al.*, 2007 ▶). At present, we have no reason to suspect that this will not also be the case for CHO cells.

We undertook the structural analysis of unliganded CTLA-4ex in order to determine, *via* comparisons with ligand-bound complexes of CTLA-4 (Schwartz *et al.*, 2001 ▶; Stamper *et al.*, 2001 ▶), whether conformational changes accompany ligand-induced triggering of the receptor. The unusual situation arose that details of the crystallization of bacterially expressed human CTLA-4ex have been reported (Chang *et al.*, 2000 ▶), but the structure was not subsequently published. Similarly, Schwartz and coworkers referred in passing to having determined the structure of the apo form of CTLA-4ex in their study of the complex of CTLA-4 with domain 1 of B7-2, but did not subsequently publish their observations (Schwartz *et al.*, 2001 ▶). We found that apo CTLA-4 monomers expressed in bacteria according to the method of Chang *et al.* (2000 ▶) formed strand-exchanged dimers during crystallization (Sonnen *et al.*, 2010 ▶). Mammalian cell-expressed material, on the other hand, yielded very high-quality crystals of an apo CTLA-4 homodimer lacking the strand exchange and is, we assume, natively folded owing to its similarities to ligand-bound CTLA-4 (Schwartz *et al.*, 2001 ▶; Stamper *et al.*, 2001 ▶). The new structure of the apo form of CTLA-4 produced as described here confirms that conformational rearrangements do not accompany ligand binding by the CTLA-4 homodimer (Yu *et al.*, 2010 ▶). These observations place additional constraints on the mechanism of signalling by receptors dependent on extrinsic tyrosine kinases such as CTLA-4 and the T-cell receptor.

## Supplementary Material

Supplementary material file. DOI: 10.1107/S1744309111017672/bw5387sup1.pdf

## Figures and Tables

**Table 1:** Data-collection and processing statistics Values in parentheses are for the highest resolution shell.

No. of crystals	1
Beamline	I04
Wavelength (Å)	0.9697
Detector	ADSC Q315r
Crystal-to-detector distance (mm)	270
Rotation range per image (°)	0.75
Total rotation range (°)	264.75
Exposure time per image (s)	0.8
Resolution range (Å)	25–1.8 (2.0–1.8)
Space group	*P*2_1_2_1_2_1_
Unit-cell parameters (Å)	*a* = 43.9, *b* = 51.5, *c* = 102.9
Mosaicity (°)	0.291
Total No. of measured intensities	211670
Unique reflections	22222 (5907)
Multiplicity	9.52 (8.23)
Mean *I*/σ(*I*)	10.33 (3.19)
Completeness (%)	99.7 (99.4)
*R*_merge_ (%)†	11.1 (33.8)
*R*_meas_ or *R*_r.i.m._ (%)	18.3 (70.8)
Overall *B* factor from Wilson plot (Å^2^)	36.803

† *R*_merge_ = 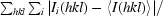 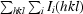, where *I_i_*(*hkl*) is the *i*th observation of reflection *hkl* and 〈*I*(*hkl*)〉 is the weighted average intensity for all observations *l* of reflection *hkl*.

## Footnotes

1 Supplementary material has been deposited in the IUCr electronic archive (Reference: BW5387).
