# Supplementary material for: Use of the α-mannosidase I inhibitor kifunensine allows the crystallization of apo CTLA-4 homodimer produced in long-term cultures of Chinese hamster ovary cells
Source: Acta Crystallogr Sect F Struct Biol Cryst Commun. 2011 Jun 30;67(Pt 7):785–9. doi: 10.1107/S1744309111017672 (PMC3144796; doi:10.1107/S1744309111017672)
Supplement: Supplementary file 1 [file f-67-00785-sup1.pdf]

Supplementary Figure

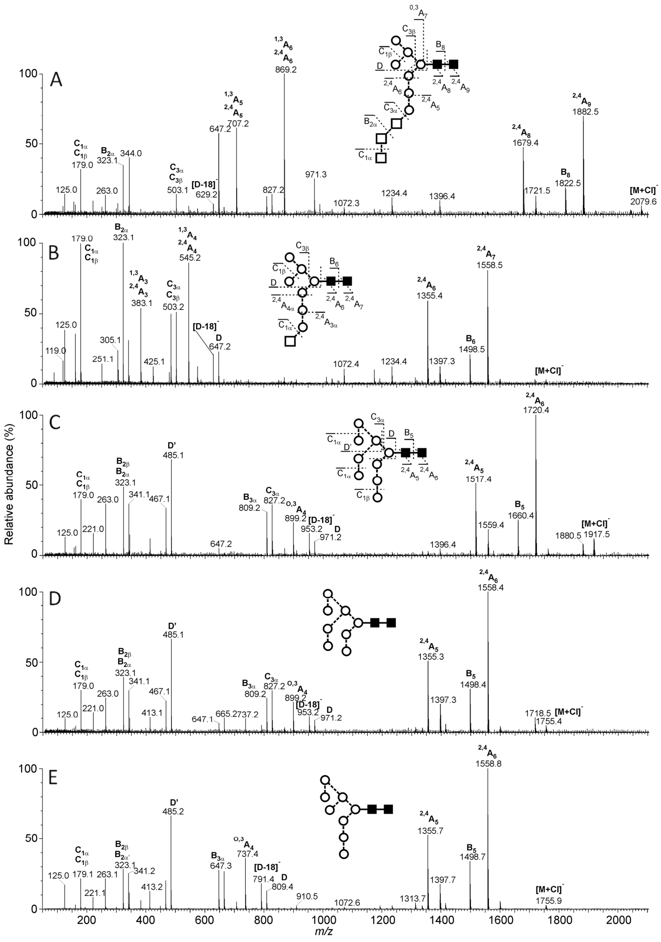

Figure S1

CID spectra of [M+Cl]$^-$ ions of the N-linked glycans from CTLA-4ex. **(A)** Hex$_{10}$HexNAc$_2$ from the glycans from CTLA-4ex expressed in CHO-K1 cells with 1.5mM NB-DNJ; **(B)** Hex$_8$HexNAc$_2$ from the glycans from CTLA-4ex expressed in CHO Lec3.2.8.1 cells with 0.5mM NB-DNJ; **(C)** Hex$_9$HexNAc$_2$ from the glycans from CTLA-4ex expressed in CHO-K1 cells with 10μM kifunensine; **(D)** Hex$_8$HexNAc$_2$ from the glycans from CTLA-4ex expressed in CHO-K1 cells with 10μM kifunensine; and **(E)** a D1,D3-Man$_8$GlcNAc$_2$ standard. Symbols as in Figure 2, main text.
